# Insight into the Effect of Glycerol on Dielectric Relaxation and Transport Properties of Potassium-Ion-Conducting Solid Biopolymer Electrolytes for Application in Solid-State Electrochemical Double-Layer Capacitor

**DOI:** 10.3390/molecules28083461

**Published:** 2023-04-14

**Authors:** Abdullahi Abbas Adam, Hassan Soleimani, John Ojur Dennis, Osamah A. Aldaghri, Ahmed Alsadig, Khalid Hassan Ibnaouf, Bashir Abubakar Abdulkadir, Ismael Abdalla Wadi, Vipin Cyriac, Muhammad Fadhlullah Bin Abd. Shukur

**Affiliations:** 1 Department of Fundamental and Applied Sciences, Universiti Teknologi PETRONAS, Seri Iskandar 32610, Perak, Malaysia; hassan.soleimani@utp.edu.my (H.S.); jdennis100@gmail.com (J.O.D.); mfadhlullah.ashukur@utp.edu.my (M.F.B.A.S.); 2 Centre of Innovative Nanoscience and Nanotechnology (COINN), Universiti Teknologi PETRONAS, Seri Iskandar 32610, Perak, Malaysia; 3 Department of Physics, Al-Qalam University Katsina, Katsina 820252, Nigeria; 4 Department of Physics, College of Science, Imam Mohammad Ibn Saud Islamic University (IMSIU), Riyadh 13318, Saudi Arabia; khiahmed@imamu.edu.sa; 5 CNR Nanotec, University Campus Ecotekne, 73100 Lecce, LE, Italy; ahmed.alsadig@nanotec.cnr.it; 6 Department of Chemistry, Gombe State University, Gombe 760214, Nigeria; abubakarbashir150@gmail.com; 7 Preparatory Year Unit, Prince Sattam Bin Abdulaziz University, Alkharj 16273, Saudi Arabia; i.a.wadi101@gmail.com; 8 Physics Department, Faculty of Education, University of Nyala, Nyala P.O. Box 155, Sudan; 9 Department of Physics, Manipal Institute of Technology, Manipal Academy of Higher Education, Manipal 576104, Karnataka, India; vipin.cyriac@learner.manipal.edu

**Keywords:** solid biopolymer electrolyte—SBE, glycerol, electrochemical double-layer capacitor—EDLC, cyclic voltammetry—CV

## Abstract

The increased interest in the transition from liquid to solid polymer electrolytes (SPEs) has driven enormous research in the area polymer electrolyte technology. Solid biopolymer electrolytes (SBEs) are a special class of SPEs that are obtained from natural polymers. Recently, SBEs have been generating much attention because they are simple, inexpensive, and environmentally friendly. In this work, SBEs based on glycerol-plasticized methylcellulose/pectin/potassium phosphate (MC/PC/K_3_PO_4_) are investigated for their potential application in an electrochemical double-layer capacitor (EDLC). The structural, electrical, thermal, dielectric, and energy moduli of the SBEs were analyzed via X-ray diffractometry (XRD), Fourier transforms infrared spectroscopy (FTIR), electrochemical impedance spectroscopy (EIS), transference number measurement (TNM), and linear sweep voltammetry (LSV). The plasticizing effect of glycerol in the MC/PC/K_3_PO_4_/glycerol system was confirmed by the change in the intensity of the samples’ FTIR absorption bands. The broadening of the XRD peaks demonstrates that the amorphous component of SBEs increases with increasing glycerol concentration, while EIS plots demonstrate an increase in ionic conductivity with increasing plasticizer content owing to the formation of charge-transfer complexes and the expansion of amorphous domains in polymer electrolytes (PEs). The sample containing 50% glycerol has a maximal ionic conductivity of about 7.5 × 10^−4^ scm^−1^, a broad potential window of 3.99 V, and a cation transference number of 0.959 at room temperature. Using the cyclic voltammetry (CV) test, the EDLC constructed from the sample with the highest conductivity revealed a capacitive characteristic. At 5 mVs^−1^, a leaf-shaped profile with a specific capacitance of 57.14 Fg^−1^ was measured based on the CV data.

## 1. Introduction

We cannot imagine a world without portable technological gadgets for our everyday activities since they have unparalleled significance. However, the operation of these gadgets depends on their capacity to store energy effectively and to deliver power on demand. For instance, lithium-ion batteries (LIBs) are the most prominent energy-storing devices because they have an appreciably high energy density of around 150 to 200 Wh/Kg [1,2]. For this reason, LIBs are commonly used in hand-held electronic gadgets as well as aircraft and other vehicles that traditionally run on fossil fuels [3,4,5]. However, the low power density of LIBs is a disadvantage in the event of a sudden surge in demand for high-power devices. Additionally, LIBs have several significant disadvantages, including the risk of explosions and decreased battery life. Furthermore, the high cost of lithium and its toxic nature also poses a challenge to the environmental sustainability of LIBs’ electrolytes [6,7].

To a greater extent, the architecture of many LIBs and other portable devices such as rechargeable batteries and supercapacitors means they are designed to operate on liquid electrolytes (LEs). However, liquid electrolytes are prone to pressure distortion and leakages, resulting in the release of hazardous compounds [1,8]. To minimize the reliance on LEs, researchers are currently putting more effort into producing solid polymer electrolytes (SPEs) because these solvent-free and non-flammable electrolytes are straightforward to deal with and can be solution-cast [9]. Furthermore, SPEs significantly minimize the risk of explosions in batteries; plus, the elimination of the need for separators and their cheaper manufacturing costs are other benefits of employing them in SCs [10,11]. However, the SPEs’ semicrystalline form results in limited ionic conductivity, thereby restricting their potential applications in many commercial devices [12]. Polymer blending (also called polyblend) is a successful method of increasing the amorphous state of SPEs and, as a result, makes it simpler for ions to migrate through the system, thus improving the SPE’s electrochemical performance [13,14,15]. Another important property of a polyblend system is that one of the polymers functions is as a natural plasticizer, enhancing the amorphous phase of the blend system [16].

Methylcellulose (MC) is a common biopolymer synthesized by substituting the OH groups of cellulose with methyl groups [17,18,19]. Thus, MC contains multiple hydrophobic methoxy groups connected to its hydrophilic polysaccharide backbone. For this reason, it has been widely employed as a polymer host in many SPEs [20,21,22]. The presence of oxygen atoms with lone pairs of electrons in MC also serve as interaction sites for the cations from the salt, which results in the formation of polymer–salt complexes. However, SPEs based on MC alone are faced with low ionic conductivity, hence the need for blending MC with another polymer [12,23]. Pectin (PC) is another biopolymer generated from plants and it is biodegradable and eco-friendly. Due to its small size and structural characteristics, this polymer offers a habitat for ions, enabling them to find their way inside the polymer much more easily than many other polymers with bigger diameters and larger molecules [24,25]. Aside from the natural abundance of both MC and PC, their biodegradability, low cost, water-solubility, biocompatibility, and presence of several OH groups are of special interest in numerous areas. Therefore, a polyblend comprising MC and PC as host materials can serve as a sustainable SBE for energy storage applications.

The mechanism of ion transportation in PEs transpires within the amorphous zone via the polymer chain’s segmental motion as well as ion hopping through the polymer chain. Therefore, the incorporation of salt into a polymer matrix is believed to increase ion permeability as well as ion transportation [26]. In comparison to lithium salts, which have been predominantly used in PE applications, potassium salts provide significant advantages for prospective use in SPEs. To mention a few of these advantages, lithium ions (Li^+^) have lower mobility in PEs than potassium ions (K^+^), which is a consequence of the polymeric matrix incorporating or trapping smaller cations such as Li^+^. Additionally, K^+^ is a more rapidly conducting ion inside various types of crystalline and amorphous materials compared to Li^+^ [27]. Furthermore, Li^+^ is electrostatically more attracted to polar groups of the host polymer than K^+^ is. This implies that Li^+^ transport within the PE requires more activation energy than K^+^ [28,29,30]. Thus, we presume that the addition of potassium salt to a polymeric system will significantly increase the electrochemical performance of the composite polymer electrolyte (CPE) system.

The primary goal of this work was to synthesize an all-natural biopolymer-based solid biopolymer electrolyte (SBE) from an MC and PC blend complex with K_3_PO_4_ and glycerol via an ultrasonication-assisted solution cast approach. A glycerol plasticizer was added to the SBE to increase the system’s conductivity and potential stability. The major purpose of ultrasonic treatment is to completely homogenize the precursor solution and enhance the miscibility of the PC/MC blend with K_3_PO_4_, and glycerol as ultrasonication is often used to ensure the complete dissociation of salt clusters inside the precursor solution [18]. Electrochemical tests on the samples were carried out utilizing complex impedance spectroscopy, linear sweep voltammetry, and transference number measurement. Additionally, physical characterizations were conducted using XRD, FTIR, and DSC, respectively. Under ambient temperature, the SBE’s optimal electrochemical performance of 7.46 × 10^−4^ scm^−1^, 3.99 V, and 0.959 ion transport number demonstrates its potential for electrochemical applications (mainly EDLC). This work, therefore, investigates the influence of glycerol on the dielectric and EDLC characteristics of PC/MC/K_3_PO_4-_based SBEs for potential application in symmetric supercapacitors.

## 2. Results and Discussion

### 2.1. Characterization of SBEs

#### 2.1.1. FTIR Analysis

A straightforward method for detecting chemical interactions in SPEs is to use FTIR spectroscopy to monitor the band shifts of specific functional groups. In an ideal situation, the salt’s cations would form complexes with the polymer’s polar groups inside the polymer matrix, leading to the formation of polymer–salt complexes [31]. In this study, FTIR analysis was performed on the SBEs to analyze the interaction of MC/PC/K_3_PO_4_ with glycerol by observing the band shifts of each functional group or the appearance/disappearance of the IR spectrum band of the parent polymers [12,21,32].

Moreover, glycerol is considered to be a reagent that might speed up the dissociation of the K_3_PO_4_ ionic dopants, resulting in a higher dissociation of K^+^ towards the MC/PC mixture. In this system, it is said that glycerol creates a network with a shorter path, enabling K^+^ to cross and pass each site with greater ease. During the conduction phase, K^+^ formed weak connections with the oxygen atoms of glycerol before jumping to the C=O of the glycerol-plasticized SBE. Additionally, the effective hop distance decreased, resulting in a lower energy threshold [33]. Consequently, it is predicted that the addition of glycerol will modify the IR spectra of the existing SBE, increasing ionic conduction.

**Figure 1:**
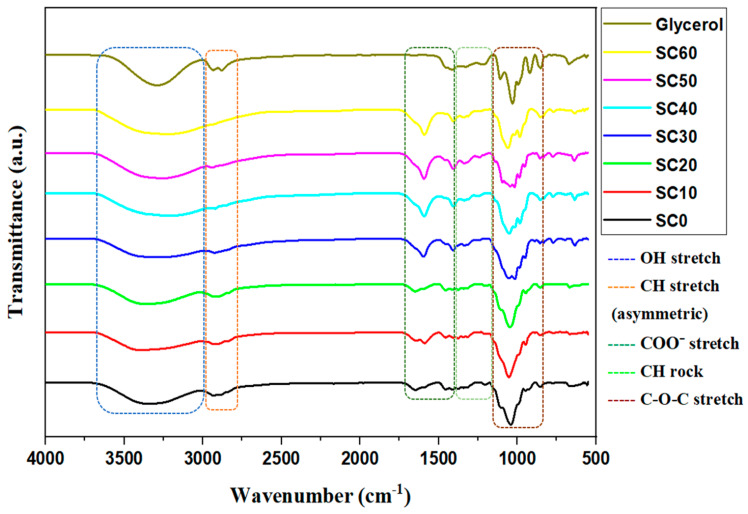
FTIR of unplasticized and glycerol-plasticized MC/PC/K_3_PO_4_ SBEs.

- According to a previous study [34], the IR spectra of glycerol consist of a broad absorption band situated at 3282 cm^−1^ which belongs to the O-H stretch and a tiny doublet at 2932 cm^−1^ due to the asymmetric stretch of C-H. Other prominent peaks of pure glycerol include C-O-H around 1414 cm^−1^ and the C-O stretch around 1029 cm^−1^ [34,35]. Very closely, the results reported in this study show that the O-H and C-H bands of glycerol appear at the vicinity of 3275–3285 cm^−1^ and 2929–2937 cm^−1^, respectively. Similarly, the C-O-H and the C-O stretching vibrations occur at 1420 and 1033 cm^−1^, respectively.
- Compared to the IR spectra of MC/PC and MC/PC/K_3_PO_4_ reported by Adam et al. [2], the influence of glycerol on the complexation of MC/PC with K_3_PO_4_ is confirmed by the alteration of the IR peaks as presented in Figure 1. As seen, the predominant OH stretch of 50 wt.% MC/PC/K_3_PO_4_ (SC0) shifted from 3354 cm^−1^ to around 3423 cm^−1^ for the glycerol-plasticized samples. The prominence of the O-H band peaks indicates complexation within the electrolyte, which promotes the dissociation of ionic species, thereby raising the ionic conductivity. Similarly to the report of Gupta and Varshney [36], the shift in the IR band assignment of the polymer–salt complex is attributed to the interplay between polymers’ (here, MC and PC) segmental motion, as well as the ion (here, K^+^)-hopping mechanism.
- In K_3_PO_4_, all potassium atoms are bonded individually. The loosely bonded potassium atom dissociates easily from the parent compound to form K^+^ and migrates from one point to another. This ion is thus responsible for conduction within the polymer matrix. The addition of glycerol in SBEs creates more pathways for potassium ion mobility and also facilitates the dissociation of ion aggregates from the salt, thereby increasing the concentration of conduction ions [31]. This is evident in the IR band shift observed for the OH stretch of SC50. Similar observations are seen in other band assignments of all of the glycerol-plasticized samples.
- At 2850 cm^−1^, the C-H asymmetrical stretching of the SBE is highly noticeable. The prominence of this peak grew as the concentration of glycerol rose, suggesting the complex evolution of glycerol in SBE. Similarly to this work, the shift of the C-H peak according to Aziz et al. [37] demonstrates the formation of a complex interaction between the CS-MC-NH_4_SCN system and the glycerol plasticizer.
- The COO^−^ stretch, C-H rock, and C-O-C stretch in the unplasticized sample (SC0) may be found around 1665 cm^−1^, 1438 cm^−1^, and 1014 cm^−1^, respectively. These peaks were altered by the inclusion of glycerol, with a minor shift noted in each instance as the glycerol content increased. The observed IR peak shift indicates that glycerol content influences the interaction of the polymer–salt complex [38]. The inclusion of glycerol causes more salts to dissolve into free ions (due to its high dielectric constant), resulting in more ions interacting with oxygen atoms in the polymer–salt plasticizer system. Therefore, all changes observed in the FTIR spectra confirm the complexation of MC, PC, K_3_PO_4_, and glycerol.

#### 2.1.2. XRD Studies

**Figure 2:**
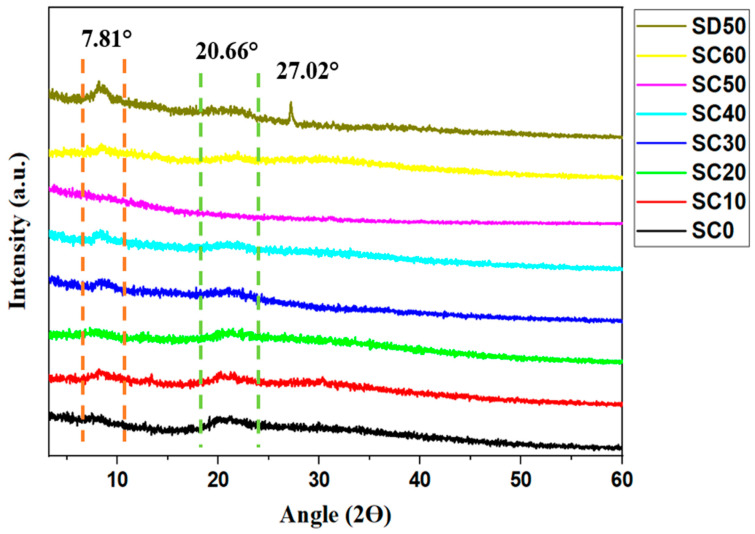
XRD patterns of unplasticized and glycerol-plasticized MC/PC/K_3_PO_4_ SBEs.

The amorphous structures of synthesized SBEs were examined using XRD to investigate their crystallinity and to support the formation complexes between the host polymers, K_3_PO_4_ salt, and glycerol plasticizer (as shown via FTIR analysis). The influence of glycerol on the polymer–salt system can be observed in Figure 2. As shown, the crystalline behavior of the SBE decreased upon dispersion of glycerol in the MC/PC/K_3_PO_4_ system. Possibly, this could be due to the interactions of the MC/PC host with K_3_PO_4_ and glycerol, thereby increasing the segmental motion of the SBE system. Furthermore, DSC analysis from our previous study [2] confirmed that the incorporation of salt and plasticizer resulted in decreased T_g_ due to improved segmental flexibility. Here, the crystallinity of the unplasticized SBE (SC0) decreases with a rise in the concentration of the plasticizer in the system until 50 wt.% (SC10–SC50). These findings imply that complexation between MC/PC, K_3_PO_4_, and glycerol improves the amorphous part of the polymer matrix, thus resulting in increased cation concentration and mobility throughout the amorphous regions of the polymer matrix. Complexation here occurs when free K^+^ is dissociated from K_3_PO_4_ salt to form cationic coordination with the oxygen atoms in the MC/PC polymer chain backbone’s OH group through the Lewis acid–base interaction (i.e., K^+^—OH) [1]. Consequently, the amorphous structure of the electrolyte results in better conductivity via increasing ion diffusivity in the electrolyte.

Since plasticizers assist in the dispersion of excess salt in the polymer matrix, we tried to investigate the influence of glycerol alone on the crystalline behavior of the MC/PC/K_3_PO_4_ system. This sample was analyzed and compared with the optimum MC/PC/K_3_PO_4_/glycerol sample (SC50). This new sample was tagged SD50 since it was comprised of 50 wt.% glycerol incorporated into the MC/PC system. As can be seen in Figure 2, the incorporation of glycerol alone showed a decrease in the MC/PC’s broad peak around 2Ɵ = 20.66°. However, excess glycerol increased the semicrystalline peak found at around 2Ɵ = 7.81°. According to an artificial intelligence algorithm study reported by Adam et al. [17], the individual effect of the plasticizer on the polymer host’s ionic conductivity is less pronounced compared to the salt’s effect. This is because the plasticizer mainly facilitates the easy channeling of salt ions within the polymer matrix in a polymer–salt complex. Furthermore, the new peak seen at 27.02° of SD50 can be attributed to excess glycerol in the MC/PC polymer matrix. Adam et al. [17] reported that a glycerol peak emerged at 26.9° when the prepared SPE was incorporated with excess glycerol. This is because, with excess plasticizer content, the plasticizer begins to replace the host polymer in the polymer backbone [2].

To further validate the influence of glycerol on the amorphous behavior of the produced samples, the degree of crystallinity ($X_{c}$) was measured for each sample using the Gaussian approach, shown by Equation (1) [12]:

(1) $$X_{c}=\frac{A_{c}}{A_{t}}\times 100\%$$

where $A_{c}$ represents the crystalline peak area and $A_{t}$ is the total hump. As shown in Table 1, the degree of crystallinity was seen to decrease with the incorporation of glycerol until the concentration exceeded 40 wt.%. The reduction in crystallinity and the rise in amorphousness generated by the addition of plasticizer are attributable to the increase in the segmental flexibility of the polymer chain and the creation of K^+^ pathways for easy ion hopping from one coordination site to the other. This is possible because the presence of glycerol molecules decreases the inter-polymer and intra-polymer hopping distances [17,31]. The trend of $X_{c}$ resembles the pattern of peaks, as seen in Figure 2.

### 2.2. Electrochemical Studies of SBEs

#### 2.2.1. EIS Studies

The impedance measurements of the series of polyblends were carried out via EIS at room temperature with the help of the AUTOLAB/AUT51018 in the frequency range between 10^5^ and 10^−2^ Hz. The complicated impedance measurements were used to estimate the conductivity values of the mixtures of MC and PC in varied proportions, according to the complex impedance (*Z_r_* and *Z_i_*) plot shown in Figure S1. The ionic conductivity of each sample was determined using Equation (2) [18]:

(2) $$\sigma =\frac{d}{R_{b}A}$$

The variables *d* and *A* in the above equation, respectively, represent the SBE’s thickness and the area of contact between the electrodes and the electrolytes. Assumably, plasticizers potentially enhance the amorphous characteristics of the PEs, as previously shown by XRD patterns (Figure 2). This consequently improves the ionic conductivity of the system by stimulating ion hopping within the system [38]. In this work, the plasticization of glycerol causes greater salt dissociation and the creation of conducting ion transfer channels within the polymer. This gives rise to an increase in the total conductivity of the SBE. Due to glycerol’s high dielectric constant, it assists in reducing the electrostatic attraction between the potassium salt cations and anions, resulting in the release of a large number of mobile conducting ions (here, K^+^) [39].

**Figure 3:**
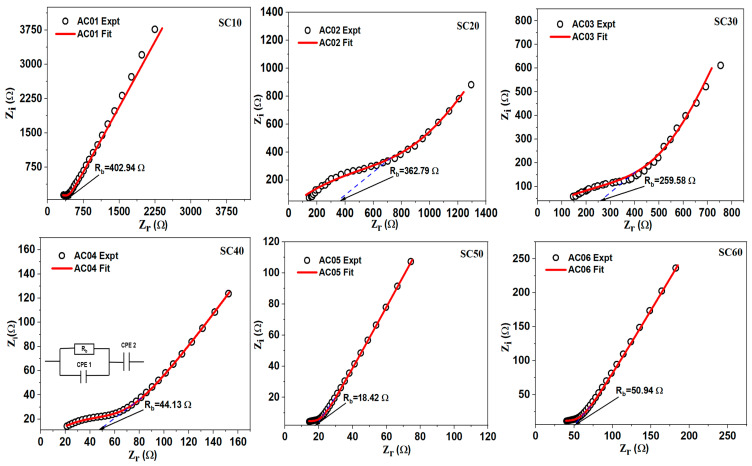
EIS plots showing the bulk resistance of MC/PC/K_3_PO_4_/glycerol SBEs.

The Nyquist curves of the glycerol-plasticized samples are presented in Figure S1, while the corresponding EEC simulations are shown in Figure 3. As can be seen in the Nyquist plots, each curve consists of a small semi-circle and a long spike at high and low frequencies, respectively. This observation is a typical attribute of an EDLC [40]. The high-frequency semi-circle is caused by a parallel arrangement of bulk resistance (due to cation migration) and bulk capacitance (arising from immobilized polymer chains), while the low-frequency spike is caused by blocking electrodes [4,41,42]. At mid-frequency, “quantum mechanical tunneling (QMT)” strictly governs the conduction technique of the higher conducting sample, while “overlapping large polaron tunnelling (OLPT)” governs the process at a higher frequency [43]. The diameter of the semi-circle is seen to decrease with increasing glycerol content up to 50 wt.%, indicating a decrease in SBE’s *R_b_*. However, the semi-circle reappears, and the conductivity decreases when 60% glycerol is added. At this point in the polymer–salt complex, the host polymer is replaced by the plasticizer molecules, which causes salt recrystallization, causing a decline in the conductivity of the complex [31].

**Figure 4:**
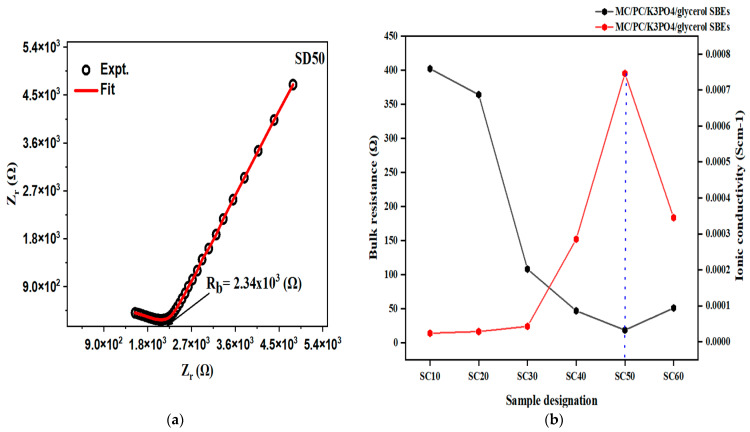
(**a**) Ionic conductivity of SD50; (**b**) ionic conductivity trend of MC/PC/K_3_PO_4_/glycerol SBEs.

When MC/PC film was doped with SD50, an ionic conductivity of 4.3 × 10^−6^ scm^−1^ was recorded (relative to 2.89 × 10^−9^ scm^−1^ of pristine MC/PC) [2]. This increase in ionic conductivity might be attributed to the creation of intermolecular coordination between H atoms from glycerol and oxygen atoms in the polymer chain, which improves the flexibility of the polymer backbone [44]. The Nyquist plot of SD50 can be seen in Figure 4a. In all the MC/PC/K_3_PO_4_/glycerol samples, however, the presence of K^+^ from K_3_PO_4_ was responsible for the appreciable rise in ionic conductivity. Furthermore, Figure 4b shows the conductivity trend (in red color) and the corresponding bulk resistance (in black color) for the effect of glycerol on the ionic conductivity of MC/PC films. Moreso, the corresponding values of the ionic conductivity computed from the Nyquist plots are shown in Table S1. As seen, the conductivity rose with increasing glycerol content until a maximum value of 7.46 × 10^−4^ scm^−1^ was attained for SC50, owing to the successful dispersion of potassium ions in the polymer matrix. For SC60, however, the decrease in conductivity was associated with the substitution of the polymer backbone due to excess glycerol, thereby demoting the polymer segmental flexibility.

#### 2.2.2. Dielectric and Energy Modulus Studies

**Figure 5:**
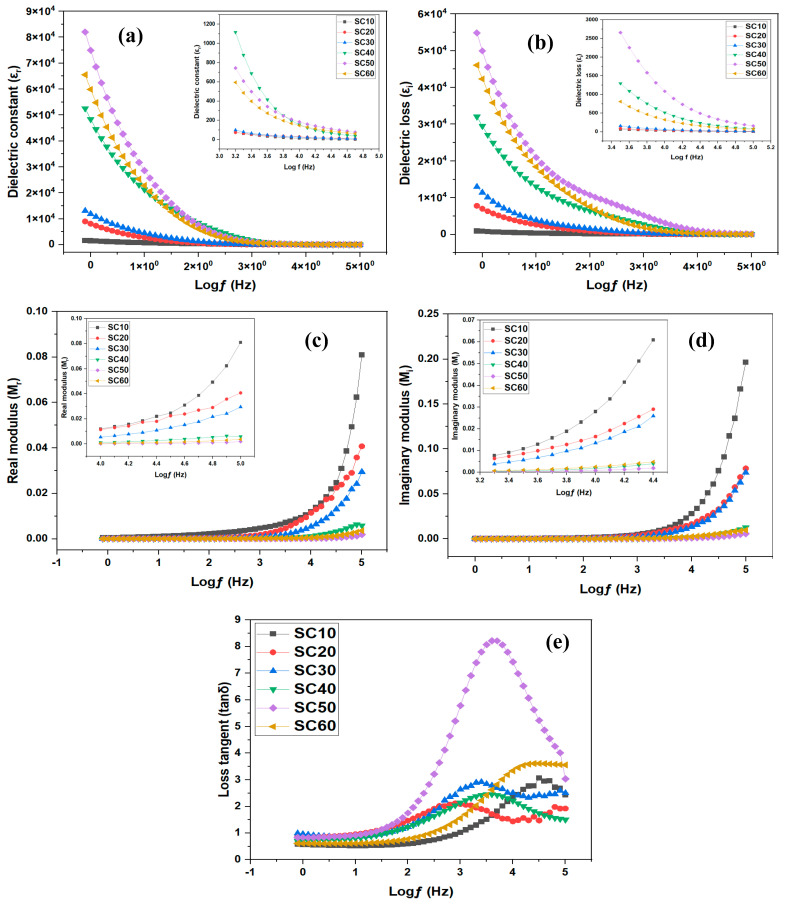
Dielectric and energy modulus of SBE: (**a**) dielectric constant, (**b**) dielectric loss, (**c**) real modulus, (**d**) imaginary modulus, and (**e**) loss tangent.

Another critical property that may be determined from the impedance tests of PEs is the material’s dielectric properties. By examining the dielectric behavior of SPE, one may be able to deduce the conductivity trend and polarization phenomena at the device’s electrode–electrolyte interface [45]. Figure 5a,b illustrates the degree to which the dielectric constant ($\varepsilon_{r})$ and dielectric loss ($\varepsilon_{i})$ are dependent on frequency for different glycerol concentrations at room temperature. The values for $\varepsilon_{r}$ and $\varepsilon_{i}$ were obtained using Equations (3) and (4), respectively [46]:

(3) $$\varepsilon_{r}=\frac{Z_{i}}{\omega C_{o}\left(Z_{r}^{2}-Z_{i}^{2}\right)}$$

(4) $$\varepsilon_{i}=\frac{Z_{r}}{\omega C_{o}\left(Z_{r}^{2}-Z_{i}^{2}\right)}$$

*Z_i_* and *Z_r_* connote the imaginary and real components of the impedance, respectively, and ω=2πf, where *f* implies frequency and $C_{o}$ symbolizes the vacuum capacitance, which is given as $C_{o}=\varepsilon_{o}A/t$ (*A*, $\varepsilon_{o}$, and *t* are the area of the sample, the permittivity of the free space, and the length of the separation, respectively).

The complex dielectric term has two parameters (i.e., $\varepsilon_{r}$ and $\varepsilon_{i})$ that represent the amount of energy stored and lost in the material. As seen in Figure 5a,b, increasing glycerol content up to 50 wt.% results in higher permittivity. This is because the facilitation of dipole orientation increased with increasing glycerol concentrations from 10 wt.% to 50 wt.%. Since more salt was being dissolved and dissociated, the density of the charge carriers increased as well. However, the dielectric constant and dielectric loss dropped dramatically when glycerol concentrations rose to 60 wt.%, because of the blocking action at the electrode/electrolyte interface [9]. This result is consistent with the ionic conductivity studies explained earlier.

To accurately estimate the conductivity behaviors of the glycerol-plasticized SBEs, it is helpful to first have a thorough grasp of the electrical characteristics of samples. At low frequencies, it is easy to see that $\varepsilon_{r}$ (Figure 5a) is much larger than $\varepsilon_{i}$ (Figure 5b). Possible explanations for this could be the dramatic difference in free-charge motion in the material [47]. A peak in the $\varepsilon_{i}$ spectrum of each sample was also seen. This indicates that dielectric relaxation was caused by the reorientation of dipoles in the polymer chains. However, the relaxation peaks were obscured in the $\varepsilon_{r}$ spectra as a result of the ion-coordinated movements.

The dielectric modulus is amongst the most effective instruments for elucidating conductivity relaxation processes because it identifies the bulk dielectric characteristics and cloaks out the electrode polarization effect. The modulus spectroscopy graph could be used to distinguish components that have equal resistance but vary enormously in capacitance, as well as to determine the electrode’s polarization, electrical conductivity, bulk behavior, and relaxation time [48]. Based on impedance records (*Z_r_* and *Z_i_*), the complex dielectric modulus of MC/PC/K_3_PO_4_/glycerol, comprising imaginary (*M_i_*) and real (*M_r_*) parts, was calculated using the following equations [1]:

(5) $$M_{r}=\left[\frac{\varepsilon_{r}}{\left(\varepsilon_{r}^{2}+\varepsilon_{i}^{2}\right)}\right]=\omega C_{o}Z_{i}$$

(6) $$M_{i}=\left[\frac{\varepsilon_{i}}{\left(\varepsilon_{r}^{2}+\varepsilon_{i}^{2}\right)}\right]=\omega C_{o}Z_{r}$$

The room temperature plots for *M_r_* and *M_i_* are shown in Figure 5c,d. Within the domain of low frequency, both *M_r_* and *M_i_* were seen to diminish with elongated tails, indicating that electrode polarization has a negligible influence in this range. However, *M_r_* and *M_i_*’s patterns exhibited the opposite behavior to that of $\varepsilon_{r}$ and $\varepsilon_{i}$ because *M_r_* and *M_i_* in the complex modulus are typically generated using the inverses of $\varepsilon_{r}$ and $\varepsilon_{i}$ in the complex impedance. Therefore, *M_r_* and *M_i_* describe the capacitive behavior of the SBEs. Furthermore, the conductivity relaxation of the electrolyte films is responsible for the obvious peaks observed in the *M_r_* and *M_i_* spectra of the SC10 and SC20.

The loss tangent (tan *δ*), sometimes referred to as the dissipation factor, reveals that the conduction of ions in PEs occurs through the polymer segmental motion and may be defined as the ratio of lost energy to stored energy (i.e., tan *δ* = $\varepsilon_{i}/\varepsilon_{r}$) during regular intervals of the field. Additionally, the mobility and conductivity of ions in PEs are significantly connected to the tan *δ*, since the area under the tan *δ* curve represents the number of ions that contribute to the relaxation process [49]. The fluctuation in the tan *δ* of SBEs plotted against log(ƒ) for various glycerol concentrations is shown in Figure 5e. As shown, the loss tangent increases at lower frequencies owing to the ohmic segment’s dominance over its capacitive counterpart. However, the loss tangent decreases at higher frequencies due to the capacitive segment’s rising pattern and its independence from the ohmic segment. The maximum at the characteristic maximum frequency ($\omega_{max})$ of Figure 5e suggests that conductivity relaxation has occurred. In this study, the relaxation time (*τ*) was calculated using Equation (7) [50] and the results are presented in Table 2.

(7) τ=1ωmax

In many investigations [51,52], it was discovered that increasing the dopant concentration moved the greatest peak of tan *δ* to a higher frequency range, implying a reduction in *τ*, commonly ascribed to increased ion mobility. However, we did not see such a linear change in the loss tangent peak in this study. A similar observation was reported by Cyriac et al. [50] and they attributed the non-linear change in loss tangent to anomalous variation in ion mobility. In this work, the anomalous relaxation time is supported by transport property studies (see Section 2.2.3).

#### 2.2.3. Transport Property Studies

To study transport characteristics such as carrier concentration (n), mobility (μ), and diffusion coefficient (D) as a function of glycerol content, the Nyquist plots were fitted using Equations (8) and (9), as provided by Arof et al. [53].

(8) $$Z_{r}=\frac{R+R^{2}k_{1}^{-1}\omega^{p_{1}}\cos\left(\frac{\pi p_{1}}{2}\right)}{1+2Rk_{1}^{-1}\omega^{p_{1}}\cos\left(\frac{\pi p_{1}}{2}\right)+R^{2}k_{1}^{-2}\omega^{2p_{1}}}+\frac{\cos\left(\frac{\pi p_{2}}{2}\right)}{k_{2}^{-1}\omega^{p_{2}}}$$

(9) $$Z_{i}=\frac{R^{2}k_{1}^{-1}\omega^{p_{1}}\sin\left(\frac{\pi p_{1}}{2}\right)}{1+2Rk_{1}^{-1}\omega^{p_{1}}\cos\left(\frac{\pi p_{1}}{2}\right)+R^{2}k_{1}^{-2}\omega^{2p_{1}}}+\frac{\sin\left(\frac{\pi p_{2}}{2}\right)}{k_{2}^{-1}\omega^{p_{2}}}$$

The parameter *R* represents bulk resistance, $k_{1}^{-1}$ is the geometrical capacitance of the bulk, $k_{2}^{-1}$ is the double-layer capacitance, $p_{1}$ is the ratio of the angle between the diameter of the semi-circle and the vertical axis to the right angle subtended by the real and imaginary impedance axis, and $\;p_{2}$ is a parameter that controls the degree of the tendency of the tilted spike from the $Z_{r}$ axis. The diffusion coefficient (*D*), mobility (*μ*), and number density (*n*) of the charge carriers were estimated with the following equations:

(10) $$D=\frac{e{\left(k_{2}\varepsilon_{r}\varepsilon_{0}A\right)}^{2}}{\tau_{2}}$$

(11) $$\mu =\frac{eD}{k_{B}T}$$

(12) $$n=\frac{\sigma }{\mu e}$$

Here, $\varepsilon_{r}$, $k_{B}$, *T*, and $\tau_{2}$ have their usual meanings. The variations in the parameters $k_{2}^{-1}$, D, μ, and n along with σ for SC10–SC60 are presented in Table 3.

As seen from Figure 5e, with an increase in glycerol content, there was no linear shift of the tan *δ* peak to the high-frequency side, indicating a decreased relaxation time. Since relaxation time and mobility are inversely related, the anomalous variation in μ is indeed seen in Table 3. Furthermore, it can be noted that $\sigma_{dc},\;\;\propto ,$ and $\epsilon^{\prime }$ are all proportional to n, and hence we can conclude from transport analysis that conductivity is strongly associated with the carrier concentration. As reported by Cyriac et al. [50], there is a strong correlation between the carrier density with increasing dopant concentration even when the mobility shows anomalous behavior.

#### 2.2.4. LSV and TNM Studies

**Figure 6:**
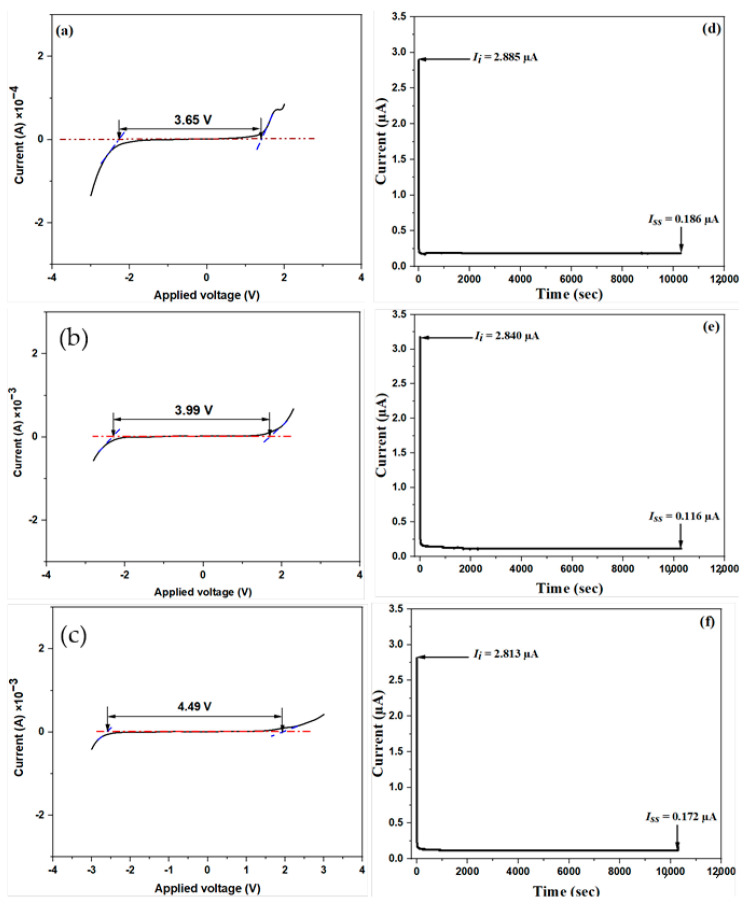
LSV curves showing the electrochemical stability window of the selected MC/PC/K_3_PO_4_-based SBEs: (**a**) unplasticized (SC0), (**b**) SC50, (**c**) SD50, and (**d**–**f**) TNM of the same samples (SC0, SC50, and SD50), showing the initial and steady-state currents.

Due to the fact that an EDLC is a high-cycle supercapacitor, it is critical to explore its operating voltage range. Before utilizing a PE in an electrochemical device, it is critical to examine its electrochemical durability and its capacity to sustain the operating voltage of the device [22,31]. In this study, the LSV analysis of the prepared SBEs was performed at a potential range of −4 to 4 V and a fixed scan rate of 10 mVs^−1^ to investigate their electrochemical stability for practical applications. The LSV plots of the unplasticized (SB50) as well as the optimum plasticized (SC50) and the reference (SD50) samples are shown in Figure 6. Furthermore, the LSV plots for the remaining plasticized samples (SC10, SC20, SC30, SC40, and SC60) are shown in Figure S2. For all of the samples, it can be discovered from the LSV plots that the applied current stayed constant even as the applied voltage rose until a maximum voltage (*V_max_*) was achieved, and at which point the current surged abruptly. This abrupt current increase is indicative of the SBE’s decomposition. From the potential window values presented in Table 4, all plasticized samples recorded a wider potential stability window relative to SC0 (3.66 V). A potential window of 3.99 V recorded by the highest conducting sample (SC50) can be taken as its electrochemical stability window, whereas the decomposition voltage for the particular SBE was obtained at 1.68 V. The potential window value obtained for this sample was found to be wider than many reported works [54,55,56,57,58,59,60,61].

According to the previous literature [62,63,64,65,66], both the plasticizer and the host polymer’s dielectric constants significantly impact electrochemical stability, thereby resulting in a larger concentration of mobile charge carriers. Thus, we may deduce that the addition of glycerol in the SBE is responsible for the rise in the electrochemical stability of the plasticized samples compared to the unplasticized sample. In support of this observation and the XRD patterns shown in Figure 2, there has also been some evidence that glycerol raised the amorphous structure of the electrolyte and perhaps boosted the mobility of ions at the polymer interface, as well as the electrolyte’s anodic stability [67,68]. Another interesting observation is that the operating potential window of SD50 (Figure 6c) was found to be 4.49 V, which is relatively wider than for both SC0 and SC50. This observation is in excellent agreement with the artificial intelligence prediction reported by Adam et al. [17]. According to their work, the plasticizer has a greater influence (compared to the salt) on the electrochemical stability window of SPEs.

Because the overall conductivity of an electrolyte is derived from the dopant salt’s ions and electrons, the TNM is a crucial type of analysis before the construction of an EDLC. In this work, TNM analysis was performed at 0.2 V to validate the polarization mechanism in the SBE. Figure 6d–f depicts the SC0, SC50, and SD50 systems’ current versus time plots, respectively. Considering SC50 (Figure 6e), at the start of the measurement, an initial high current (*I_i_*) of 2.84 μA was recorded because both ions and electrons contributed to the net current. As the analysis proceeded, the ionic blocking contrivance of the stainless-steel holder caused a decrease in the current value. At this moment, the salt cations and anions began to produce a polarization effect at the positive and negative electrodes, respectively. As this current began to normalize around 0.12 μA, a steady-state current (*I_ss_*) was attained, and complete polarization could be seen. The ion transference number (*t_ion_*) and electron transference number (*t_el_*) for SC50 were calculated as 0.959 and 0.041, respectively, using Equations (13) and (14) [2]:

(13) $$t_{ion}=\frac{I_{i}-I_{ss}}{I_{i}}$$

(14) $$t_{el}=1-t_{ion}$$

Summarily, all samples prepared in this work show extremely high potential window and cation transference number values, making them very suitable for electrochemical device applications. Furthermore, the results (ionic conductivity and electrochemical potential window) obtained in this study were compared with some reported work on glycerol-plasticized polymer-blended electrolytes, as can be seen in Table 5. From the results obtained, this work showed higher ionic conductivity and a wider potential window, making it a better candidate for electrochemical applications.

#### 2.2.5. Device Studies

##### CV Analysis

**Figure 7:**
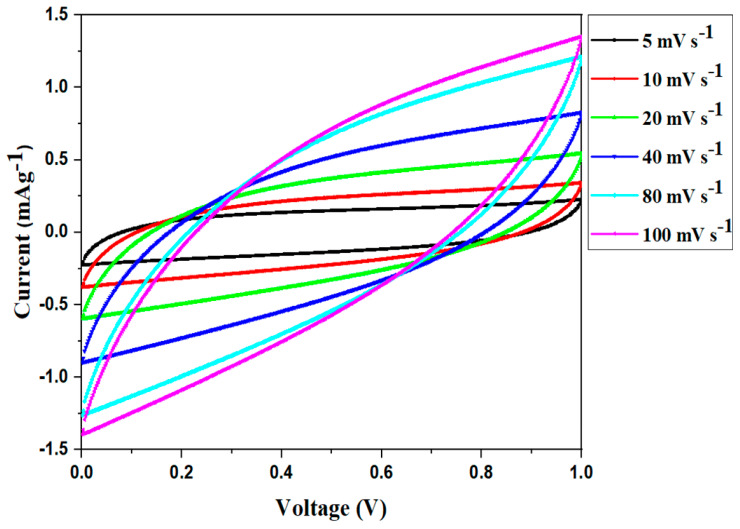
CV profile of the fabricated EDLC at different scant rates.

The consequence of different scan rates on the elementary feature of the CV portrait of SC50-based EDLC is presented in Figure 7. As can be seen in the plots, the CV cycles display a leaf-like pattern at all of the scan rates and there appears to be no redox peak over the voltage range. In contrast to the typical CV plot, which exhibits a characteristic rectangular pattern, the CV plots recorded in this research reveal a pattern that deviates from a perfect rectangle. This deviation is owing to the porous nature of the electrode’s surface, which results in changes in the internal resistance. Furthermore, such a shift in the CV response affected the EDLC’s performance and efficiency [72]. In addition, the vanishing of redox peaks in the CV profile demonstrated the existence of a charge-storage capacity through a non-faradaic mechanism, which is an essential characteristic of an ideal EDLC. Therefore, intercalation and deintercalation processes were absent [73,74].

Table 6 displays the CV values of the manufactured EDLC at different scan rates. As can be seen, the maximum value is obtained at a slow scan rate (5 mVs^−1^) and this declines noticeably as the scan rate increases. At slow scan rates, ion adsorption generates a persistent double layer of charges at the electrode–electrolyte interface [75]. A nearly ideal plateau, compared to the rest of the runs, is achieved at low scan rates due to the development of a broad diffusion layer at the interface as a consequence of ion adsorption. Owing to this action, ohmic resistance is reduced. However, a modest diffusion layer is generated at higher scan rates, leading to low specific capacitance [72].

## 3. Materials and Methods

### 3.1. Materials

MC (viscosity of 4000 cP) was obtained from Sigma Aldrich, Malaysia, whereas PC (Mw of 30,000 gmol^−1^), K_3_PO_4_ (M_w_ of 212.27 gmol^−1^), and glycerol (Mw of 92.09 gmol^−1^) were obtained from R and M Chemicals, Malaysia. All chemicals were provided by Evergreen Chemicals Supply (Shah Alam, Malaysia), and they were used without further processing. As the only solvent for the duration of the experiment, DI water was used.

### 3.2. Methodology

**Figure 8:**
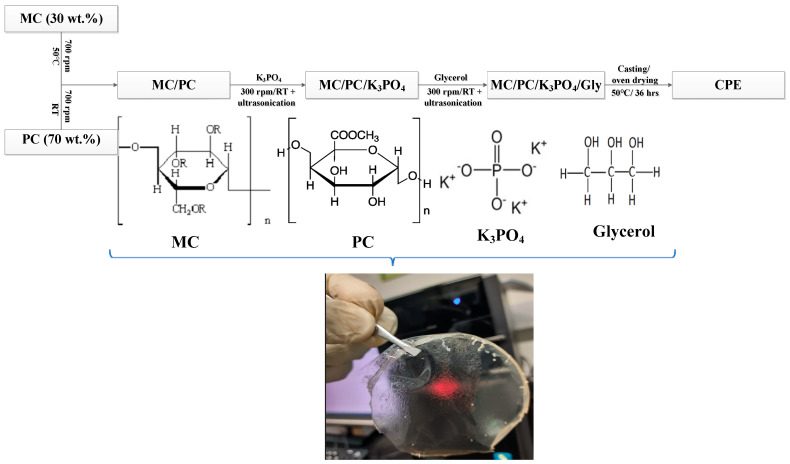
A schematic diagram describing the synthesis of SBEs.

In this study, the schematic diagram summarizing the synthesis of SBE is shown in Figure 8. This method is similar to a reported work (with slight modification) [17]. Briefly, 30 wt.% (0.3 g) of MC was dissolved in 50 mL DI water and stirred for a few hours at 50 °C. At the same time, 70 wt.% (0.7 g) of PC was dissolved in 30 mL DI water and stirred at room temperature. The two solutions (when fully dissolved) were mixed and stirred for about 1 h, followed by 30 min ultrasonication to ensure homogeneity. To the stirring solution, K_3_PO_4_ (50 wt.% corresponding to 1.0 g) was incorporated into the precursor solution and stirred further for 60 min. This sample was labeled SC0 since it contained no glycerol. Further stirring of the solution was performed at a low speed (200 rpm) to evenly disperse the samples’ constituents. The homogenous solution was allowed to settle for one more hour to remove air bubbles before being poured into Teflon Petri dishes to form the solid films (after drying). The solution drying was conducted in an oven at 50 °C for nearly 2 days to obtain the polymer dry films. The prepared films were peeled off and kept in a desiccator containing silica gel for further drying before subjecting them to characterization. To prepare the plasticized SBEs, different concentrations of glycerol (10–60 wt.%) were added to the previously stirred SC0 solution about 10 min after adding K_3_PO_4_. The prepared samples were labeled SC10–SC60 to distinguish them from the unplasticized sample. The preparation of SD50 involved the incorporation of 50 wt.% glycerol to the MC/PC solution followed by 30 min of stirring before casting and drying.

### 3.3. Characterization of Electrolyte Samples

The produced films were analyzed via Fourier transform infrared (FTIR) spectroscopy (Bruker Instruments, model Aquinox 55, Germany) in the 4000–400 cm^−1^ range using KBr pellets and a scanning resolution of 4 cm^−1^. To determine the crystal structure of the produced films, X-Ray diffraction (XRD) was performed using a 40-kV Bruker D8 Advance X-ray diffractometer with a 40-mA current and a Ni-filtered Cu K graphite monochromator (Ś = 1.5406 Å). Differential scanning calorimetry (DSC; Model DSC Q2000 V24.11, Oberkochen, Germany) was used to examine the thermal behavior of the SBEs.

### 3.4. Electrochemical Studies of Electrolyte Samples

Electrochemical studies (EIS and LSV) were used to measure the ionic conductivity, dielectric properties, and operating potential window of each SBE. The electrochemical measurements were performed at room temperature using a two-electrode setup connected to the AUTOLAB/AUT51018. The measurement was conducted in the frequency range of 10^5^–10^−2^ Hz with an applied voltage of 10 mV. Each SBE sample was held between two opposite blocks of a stainless-steel sample holder with a surface area of 3.142 cm^2^.

### 3.5. Measurement of Ion Transference Number

To investigate the major charge-carrying species, the transference number measurement (TNM) was executed on the highest conducting sample (SC50) via a digital dc power supply (V&A Instrument DP3003).

### 3.6. Dielectric and Electrical Modulus Studies of Electrolyte Samples

The Nyquist plot was used to investigate the dielectric properties and modulus formalism of the synthesized SBEs. Dielectric constant, dielectric loss, real modulus, imaginary modulus, and loss tangent were some of the dielectric parameters studied in this research.

### 3.7. EDLC Fabrication and Characterization

**Figure 9:**
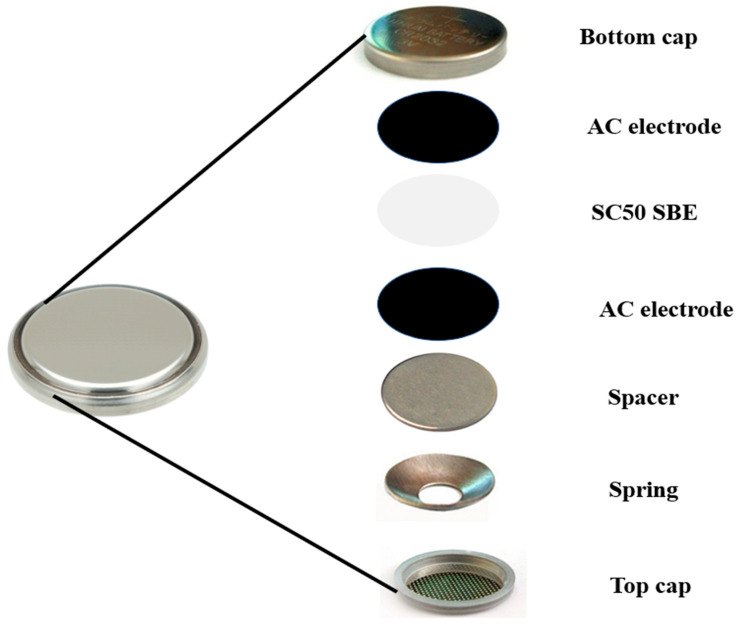
Illustration of EDLC fabrication using CR2032 coin cell assembly.

To fabricate the EDLC, two activated carbon (AC)-coated nickel foams and the optimum SBE (SC50) were cut into a circular shape to fit into a CR2032 coin cell. The AC (12.01 gmol^−1^) was procured from Sigma Aldrich, Malaysia. The SC50 sample was sandwiched between the two AC electrodes and crimped using an electric crimping machine. The configuration of the EDLC can be seen in Figure 9.

To prepare the AC electrode, 1 g polyvinylidene fluoride (PVDF) was dispersed in 30 mL N-methyl pyrrolidone (NMP) and continuously stirred at 50 °C. After about 20 min, the solution was cooled to room temperature while being continually stirred. Subsequently, 0.5 g of carbon black was gradually poured into the stirring solution at room temperature and maintained under this condition for 2 h. Finally, 6.5 g of AC was gradually added, and the mixture was further stirred until completely dissolved. The slurry was ultrasonicated for 30 min (to ensure homogeneity), and was then stirred on a hot plate at room temperature for another 1 h before being cooled to room temperature. The resulting slurry was coated on previously cleaned, pressed, and weighted nickel foams. The coated nickel foams were dried for 24 h at 50 °C before being reweighed to determine the mass of the deposited slurry. For the fabrication of the EDLC, two AC electrodes of comparable slurry masses were matched and assembled.

Cycle voltammetry (CV) was used to evaluate the electrochemical properties of the fabricated EDLC at six different scan rates: 5 mVs^−1^, 10 mVs^−1^, 20 mVs^−1^, 40 mVs^−1^, 80 mVs^−1^, and 100 mVs^−1^, in the potential range of 0 V to 1 V. The CV experiments were carried out with the use of a Potentiostat (AUTOLAB/AUT51018) equipped with Nova 2.1.4 software. The specific capacitance (*C_sp_*) of the EDLC cell was calculated using the following formula [76]:

(15) $$C_{sp}=\frac{4}{mv\left(V_{f}-V_{i}\right)}\;\int_{V_{i}}^{V_{f}}I\;\left(V\right)dV$$

where *I*(*V*)*dV* represents the CV’s area as estimated by OriginPro 2021’s program integration function. The parameters *m* and *v* represent the overall mass of active materials and the scan rate, respectively. In this work, the initial and final applied potentials (*V_i_* and *V_f_*) were maintained at 0 V and 1 V, respectively.

## 4. Conclusions

Methylcellulose/pectin-based solid biopolymer electrolytes complexed with potassium phosphate and glycerol have been propitiously prepared via the solution cast methodology. FTIR analysis confirmed the interaction of the host polymer–dopant salt (K_3_PO_4_) complex with the plasticizer (glycerol) via band shifting and the alteration of peak intensities. Furthermore, XRD studies confirmed that the incorporation of glycerol greatly improved the amorphous properties of the SBEs, thereby improving ionic conductivity. EIS analysis showed that the sample containing 50 wt.% glycerol was found to be optimum with an ionic conductivity (at room temperature) of ~7.5 × 10^−4^ scm^−1^. Furthermore, this sample displayed excellent stability with a wide potential window of 3.99 V and a high ion transference number of 0.959. The dielectric constant of the SBEs was also found to increase with increasing glycerol content up to 50 wt.%, while the complex energy modulus showed an opposite trend, indicating a negligible influence of electrode polarization at a lower frequency domain. Loss tangent analysis confirmed the ohmic segment’s dominance over its capacitive counterpart at the lower frequency region. The EDLC characteristics displayed a leaf-like CV profile with a specific capacitance of 57.14 Fg^−1^ recorded at 5 mVs^−1^. This work thus demonstrated the suitability of MC/PC/K_3_PO_4_/glycerol SBE as a new type of simple, cheap, sustainable, and highly stable SPE for EDLC applications [76].

## Figures and Tables

**Table 1:** Degree of crystallinity of MC/PC/K_3_PO_4_/glycerol SBEs.

Samples	X*_c_* (%)
SC0	20.26
SC10	19.89
SC20	19.43
SC30	19.27
SC40	19.18
SC50	18.77
SC60	19.09
SD50	20.65

**Table 2:** Relaxation time of various concentrations of glycerol-plasticized SPEs.

Glycerol Conc. (wt.%)	ƒ (Hz)	*ω* (rads^−1^)	*τ* (s)
0	31,623	1.99 × 10^5^	5.03 × 10^−6^
10	50,119	3.15 × 10^5^	3.18 × 10^−6^
20	63,096	3.96 × 10^5^	2.52 × 10^−6^
30	2511.3	1.58 × 10^5^	6.34 × 10^−5^
40	3162.3	1.99 × 10^4^	5.03 × 10^−5^
50	3981.1	2.50 × 10^4^	4.00 × 10^−5^
60	31,623	1.99 × 10^5^	5.03 × 10^−6^

**Table 3:** Summary of the parameters $k_{2}^{-1}$, *D*, *μ*, and n for the plasticized samples.

Sample	$k_{2}^{-1}\;$ (F)	σ × 10^−5^ (s/cm)	n × 10^16^ (cm^−3^)	*μ* × 10^−3^ (cm^2^ V^−1^ s^−2^)	D × 10^−4^ (cm^2^ s^−1^)
SC10	2.68 × 10^−6^	2.36	2.27	4.22	1.09
SC20	1.42 × 10^−5^	2.81	2.30	2.55	0.66
SC30	1.03 × 10^−5^	4.25	3.33	3.89	1.00
SC40	1.31 × 10^−4^	28.49	40.20	2.94	0.76
SC50	8.76 × 10^−5^	74.55	69.92	5.61	1.45
SC60	4.22 × 10^−5^	34.52	39.72	4.39	1.13

**Table 4:** Potential window values for MC/PC/K_3_PO_4_/glycerol SBEs.

Sample	Potential Window (V)
SC10	3.72
SC20	4.06
SC30	4.49
SC40	4.51
SC50	3.99
SC60	3.62

**Table 5:** Comparison of electrochemical performance of some glycerol-plasticized biopolymer-based SPEs.

Host Polymer	Salt	Ionic Conductivity (scm^−1^)	Potential Window (V)	TNM	Reference
Chitosan/Dextran	NH_4_PF_6_	3.06 × 10^−4^	1.5	0.957	[69]
Chitosan/Methylcellulose	NH_4_I	6.65 × 10^−4^	2.2	0.97	[70]
Methylcellulose/Potato Starch	NH_4_NO_3_	4.37 × 10^−5^	1.88	-	[16]
Gum Arabic/Polyvinyl Alcohol	Acetic acid	2.22 × 10^−5^	-	-	[71]
Chitosan/Dextran	NaTf	6.10 × 10^−5^	2.55	0.988	[38]
Chitosan/Methylcellulose	NH_4_NO_3_	1.31 × 10^−4^	1.87	0.933	[22]
Methylcellulose/Pectin	K_3_PO_4_	7.46 × 10^−4^	3.99	0.959	This work

**Table 6:** Variation in specific capacitance at different scan rates for the fabricated EDLC.

Scan Rates (mV s^−1^)	*C_sp_* (F g^−1^)
5	57.14
10	37.23
20	23.56
40	15.12
80	9.05
100	7.57

## Data Availability

The data will be made available by the corresponding author upon request.

## Supplementary Materials

The following supporting information can be downloaded at https://www.mdpi.com/article/10.3390/molecules28083461/s1: Figure S1: EIS plots showing the bulk resistance of MC/PC/K_3_PO_4_/glycerol SBEs; Figure S2: LSV curves showing the electrochemical stability window of remaining MC/PC/K_3_PO_4_/glycerol-based SBEs; Table S1: Ionic conductivity of MC/PC/K_3_PO_4_/glycerol SBEs.

## Sample Availability

Available on request.
