# Supplementary material for: Insight into the Effect of Glycerol on Dielectric Relaxation and Transport Properties of Potassium-Ion-Conducting Solid Biopolymer Electrolytes for Application in Solid-State Electrochemical Double-Layer Capacitor
Source: Molecules. 2023 Apr 14;28(8):3461. doi: 10.3390/molecules28083461 (PMC10146172; doi:10.3390/molecules28083461)
Supplement: Supplementary file 1 [file molecules-28-03461-s001.zip › molecules-2322893-supplementary.pdf]

Insight into the effect of glycerol on dielectric relaxation and transport properties of potassium ion-conducting solid biopolymer electrolytes for application in solid-state EDLC

Abdullahi Abbas Adam[1,2,3]*, Hassan Soleimani[1], John Ojur Dennis[1], Osamah A. Aldaghri[4,*], Ahmed Alsadig[5], Khalid Hassan Ibnaouf[4], Bashir Abubakar Abdulkadir[6], Ismael Abdalla Wadi[7,8], Vipin Cyriac[9], Muhammad Fadhlullah Bin Abd. Shukur[1,2]

[1] Department of Fundamental and Applied Sciences, Universiti Teknologi PETRONAS, Seri Iskandar 32610, Perak, Malaysia; hassan.soleimani@utp.edu.my (H.S.); mfadhlullah.ashukur@utp.edu.my (M.F.B.A.S.); jdennis100@gmail.com (J.O.D.)
[2] Centre of Innovative Nanoscience and Nanotechnology (COINN), Universiti Teknologi PETRONAS, Seri Iskandar 32610, Perak, Malaysia
[3] Department of Physics, Al-Qalam University Katsina, Katsina 820252, Nigeria
[4] Department of Physics, College of Science, Imam Mohammad Ibn Saud Islamic University (IMSIU), Riyadh 13318, Kingdom of Saudi Arabia (KSA); khiahmed@imamu.edu.sa (K.H.I.)
[5] CNR Nanotec, University Campus Ecotekne, 73100 Lecce LE, Italy; ahmed.alsadig@nanotec.cnr.it (A.A)
[6] Department of Chemistry, Gombe State University, P. M. B. 127, Gombe, Gombe State, Nigeria; abubakarbashir150@gmail.com (A.B.A.)
[7] Prince Sattam Bin Abdulaziz University, Preparatory Year Unit, Alkharj, Saudi Arabia; i.a.wadi101@gmail.com (I.A.W.)
[8] University of Nyala, Faculty of Education, Physics Department, Nyala, Sudan.
[9] Department of Physics, Manipal Institute of Technology, Manipal Academy of Higher Education, Manipal 576104, Karnataka, India; vipin.cyriac@learner.manipal.edu (V.C)

* Correspondence: abbasabdullah17@hotmail.com (A.A.A.); odaghri@imamu.edu.sa (O.A.)

Table S1. Ionic conductivity of MC/PC/K_3PO_4/glycerol SBEs

Sample	Film thickness x10^{-2} (cm)	Bulk resistance (Ω)	Ionic conductivity (Scm^{-1})
SC10	2.98	4.02×10^{2}	2.36×10^{-5}
SC20	3.19	3.63×10^{2}	2.81×10^{-5}
SC30	3.47	1.08×10^{2}	4.25×10^{-5}
SC40	4.18	4.67×10^{1}	2.85×10^{-4}
SC50	4.31	1.84×10^{1}	7.46×10^{-4}
SC60	5.52	5.09×10^{1}	3.45×10^{-4}

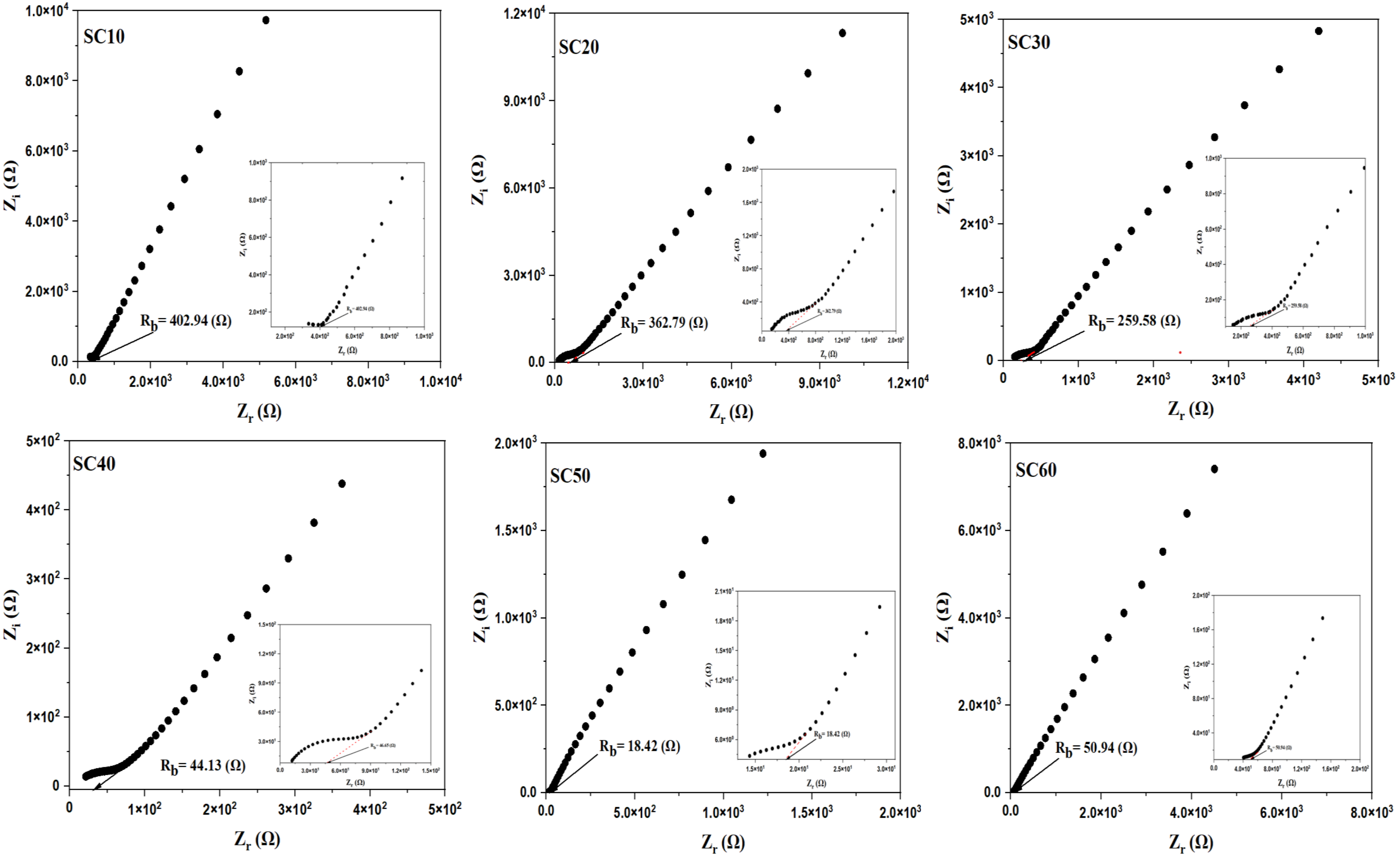

Figure S1. EIS plots showing the bulk resistance of MC/PC/K3PO4/glycerol SBEs

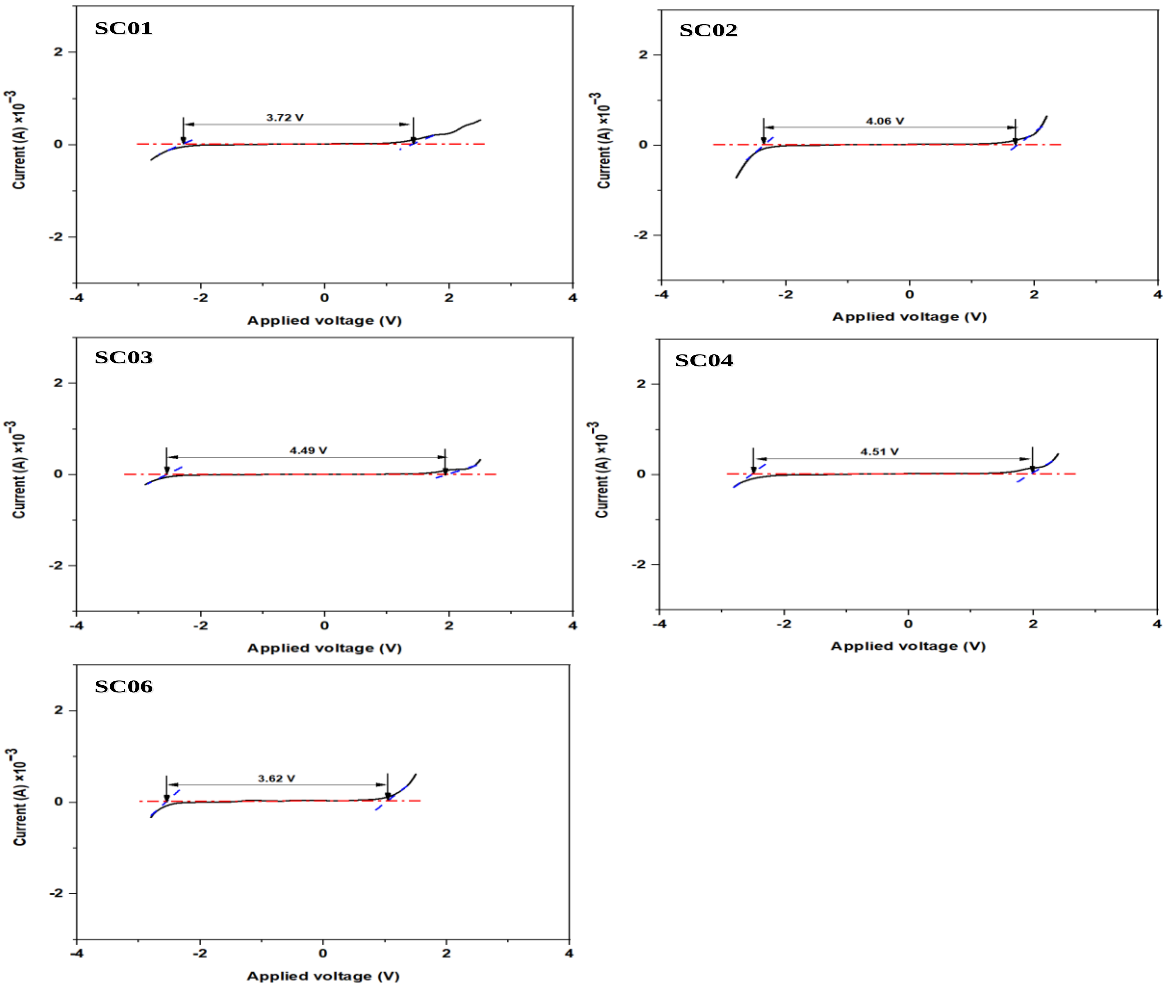

Figure S2. LSV curves showing the electrochemical stability window of remaining MC/PC/K_3PO_4/glycerol-based SBEs
